# Socioeconomic and Urban–Rural Disparities in Mammographic Tumor Burden at Breast Cancer Diagnosis: A Regional Screening Study from Eastern Romania

**DOI:** 10.3390/diagnostics16162526

**Published:** 2026-08-11

**Authors:** Tudor Gramada, Smaranda Gramada-Stefurac, Stefan Morarasu, Sorinel Lunca, Dragos Pieptu, Mihaela Elena Breaban, Gabriel Mihail Dimofte

**Affiliations:** 1 Faculty of Medicine, Grigore T. Popa University of Medicine and Pharmacy, 700115 Iasi, Romania; tudorgramada@gmail.com (T.G.); morarasu.stefan@gmail.com (S.M.); sdlunca@yahoo.com (S.L.); dpieptu@gmail.com (D.P.); gdimofte@gmail.com (G.M.D.); 2 Department of Radiology, Regional Institute of Oncology (IRO), 700483 Iasi, Romania; 3 2nd Department of Surgical Oncology, Regional Institute of Oncology (IRO), 700483 Iasi, Romania; 4 Faculty of Computer Science, “Alexandru Ioan Cuza” University of Iasi, 700483 Iasi, Romania

**Keywords:** breast cancer screening, socioeconomic disparities, rural population, mammography, tumor burden, Eastern Europe, mobile mammography, breast imaging

## Abstract

**Background/Objectives:** Breast cancer mortality remains disproportionately high in Eastern Europe, partly due to delayed diagnosis and limited access to organized screening programs. Socioeconomic deprivation and rural residence may contribute to inequalities in breast cancer detection and disease burden at presentation. This study aimed to evaluate the association between sociodemographic disparities and mammographic tumor burden at breast cancer diagnosis within a large regional screening cohort from Eastern Romania. **Methods:** This retrospective study was conducted within the ONCOFEM regional breast cancer screening initiative between August 2020 and December 2023. A total of 23,886 asymptomatic women aged 50–69 years underwent screening mammography using fixed and mobile digital mammography units. Sociodemographic variables included residential environment (urban/rural) and vulnerability status. Histopathologically confirmed malignant lesions were analyzed regarding maximum mammographic tumor dimension and focality status (unifocal, multifocal, multicentric). Statistical analysis included Mann–Whitney U tests and Chi-square/Fisher exact tests, with *p* < 0.05 considered statistically significant. **Results:** The screened population included 12,652 rural women (53.0%) and 11,234 urban women (47.0%). Urban participants demonstrated significantly higher recall rates compared with rural women (7.75% vs. 4.85%, respectively; *p* < 0.001). Similarly, the Cancer Detection Rate (CDR) was significantly higher in the urban subgroup, reaching 7.21 cancers per 1000 screened women, compared with 4.19 cancers per 1000 screened women in the rural population (*p* = 0.002). The final analytic cohort included 134 patients with histopathologically confirmed breast cancer, corresponding to 138 malignant lesions. Median tumor size was 25 mm overall. Rural and vulnerable women demonstrated numerically higher proportions of tumors exceeding 20 mm; however, no statistically significant differences were observed regarding maximum tumor dimensions according to residential environment (*p* = 0.799) or vulnerability (*p* = 0.934). Multifocal and multicentric disease represented a minority of cases, without significant associations with the sociodemographic subgroup. **Conclusions:** Although significant urban–rural differences were identified in screening performance metrics, mammographic indicators of tumor burden demonstrated relatively similar distributions across sociodemographic subgroups. The persistently high proportion of tumors exceeding 20 mm across all groups suggests a substantial burden of relatively advanced disease at diagnosis within the Romanian population. No statistically significant disparities in mammographic tumor burden were identified across the analyzed sociodemographic subgroups; however, the present study cannot determine whether the use of mobile mammography units contributed to this observation.

## 1. Introduction

Breast cancer remains one of the leading causes of cancer-related morbidity and mortality among women worldwide [1,2]. In Romania, breast cancer is the most frequently diagnosed malignancy among women, with an estimated 12,685 new cases and 3877 deaths in 2022, corresponding to age-standardized incidence and mortality rates of 69.4 and 16.6 per 100,000 women, respectively [3]. Directly comparable population-based data on tumour diameter at diagnosis across European countries remain limited, as cancer registries more frequently report disease stage rather than exact tumour size. Nevertheless, available evidence indicates that breast cancer is diagnosed at a later stage in several Eastern European countries compared with the rest of the WHO European Region [4]. These differences may partly reflect delayed diagnosis and historically limited access to organized population-based screening programs.

Mammography-based screening plays a central role in the early detection of breast cancer by enabling the identification of lesions at asymptomatic and potentially curable stages [5,6,7]. However, the effectiveness of early detection strategies is strongly influenced by social determinants of health and by the accessibility of dedicated breast imaging infrastructure. In underserved rural regions, geographic isolation, socioeconomic deprivation, reduced access to specialized medical services, lower educational status, and limited health literacy may contribute to delayed participation in screening and more advanced disease at presentation [8,9,10].

Tumor burden at diagnosis remains one of the most important prognostic indicators in breast cancer [11]. Larger tumor dimensions, as well as multifocal or multicentric disease, are frequently associated with more complex therapeutic management, higher likelihood of nodal involvement, and less favorable oncologic outcomes [11,12]. Imaging-derived tumor burden parameters may therefore provide indirect markers of delayed diagnostic access and disease severity at presentation, particularly in socially vulnerable populations.

To reduce inequalities in access to early breast cancer detection, regional screening initiatives were implemented in Romania between 2020 and 2023. An important component of these programs was the deployment of mobile mammography units designed to improve accessibility for women living in remote and underserved communities [13]. Beyond increasing participation in mammographic evaluation, these initiatives provide a valuable opportunity to investigate the relationship between sociodemographic factors and the imaging characteristics of breast cancers detected during the early implementation phase of organized screening.

Previous studies conducted in both Western and Eastern Europe have consistently demonstrated associations between socioeconomic disadvantage and more advanced breast cancer presentation at diagnosis. Nevertheless, relatively limited data are available regarding the relationship between socioeconomic vulnerability and imaging-based tumor burden parameters within the Romanian population. Furthermore, given the relatively recent implementation of regional breast cancer screening initiatives in Romania, many cancers detected during early screening rounds may still reflect prevalent rather than incident disease burden [8,9,10].

The primary objective of this study was to evaluate the association between sociodemographic disparities and mammographic tumor burden at breast cancer diagnosis within a large regional screening cohort comprising 23,886 women screened between 2020 and 2023 in Eastern Romania. Specifically, we investigated whether women from rural or socially vulnerable populations presented with larger tumors and higher rates of multifocal or multicentric disease compared with women from urban or non-vulnerable groups.

## 2. Materials and Methods

### 2.1. Ethical Considerations

All procedures performed in this study were conducted in accordance with the ethical standards applicable in Romania for this type of research and complied with the principles of the Declaration of Helsinki. Ethical approval was obtained from the Research Ethics Committee of the “Grigore T. Popa” University of Medicine and Pharmacy, Iași, Romania (Approval No. 750/15 April 2026). Written informed consent was obtained from all participants prior to inclusion in the screening program. Given the retrospective design and the complete anonymization of the dataset, the requirement for individual informed consent for participation was waived by the local Ethics Committee, which reviewed and approved the study. Therefore, informed consent for participation was not required, in accordance with the Ethics Committee approval.

### 2.2. Study Design and Population

This retrospective study was conducted within the framework of the ONCOFEM regional breast cancer screening initiative implemented in North-Eastern and South-Eastern Romania between August 2020 and December 2023. The program used an invitation-based, capacity-limited recruitment approach. Eligible asymptomatic women aged 50–69 years were identified locally and invited by community health nurses. However, invitations were not sent to all eligible women within a predefined geographic population, and no random or exhaustive population sampling framework was applied. The number of women invited was determined by the operational capacity of the program, particularly the availability of a single mobile mammography unit and the available nursing and medical staff. Consequently, the screened cohort should be interpreted as a capacity-limited invited regional cohort rather than as complete population coverage, and a population-level uptake rate could not be estimated. A complete centralized record of all invitations issued was not available; therefore, an invitation-based response rate could not be calculated. The North-Eastern and South-Eastern regions were both included in the programme; however, participation was higher in the North-East. This difference primarily reflected logistical factors. The Regional Institute of Oncology Iași, which coordinated the programme and hosted the fixed screening facilities, is located in North-Eastern Romania. Increasing distance from the coordinating centre, particularly for South-Eastern localities, required longer travel and greater logistical resources for deployment of the single mobile mammography unit and the available screening staff. As a result, screening activities were more frequently carried out in areas closer to the coordinating centre.

**Figure 1:**
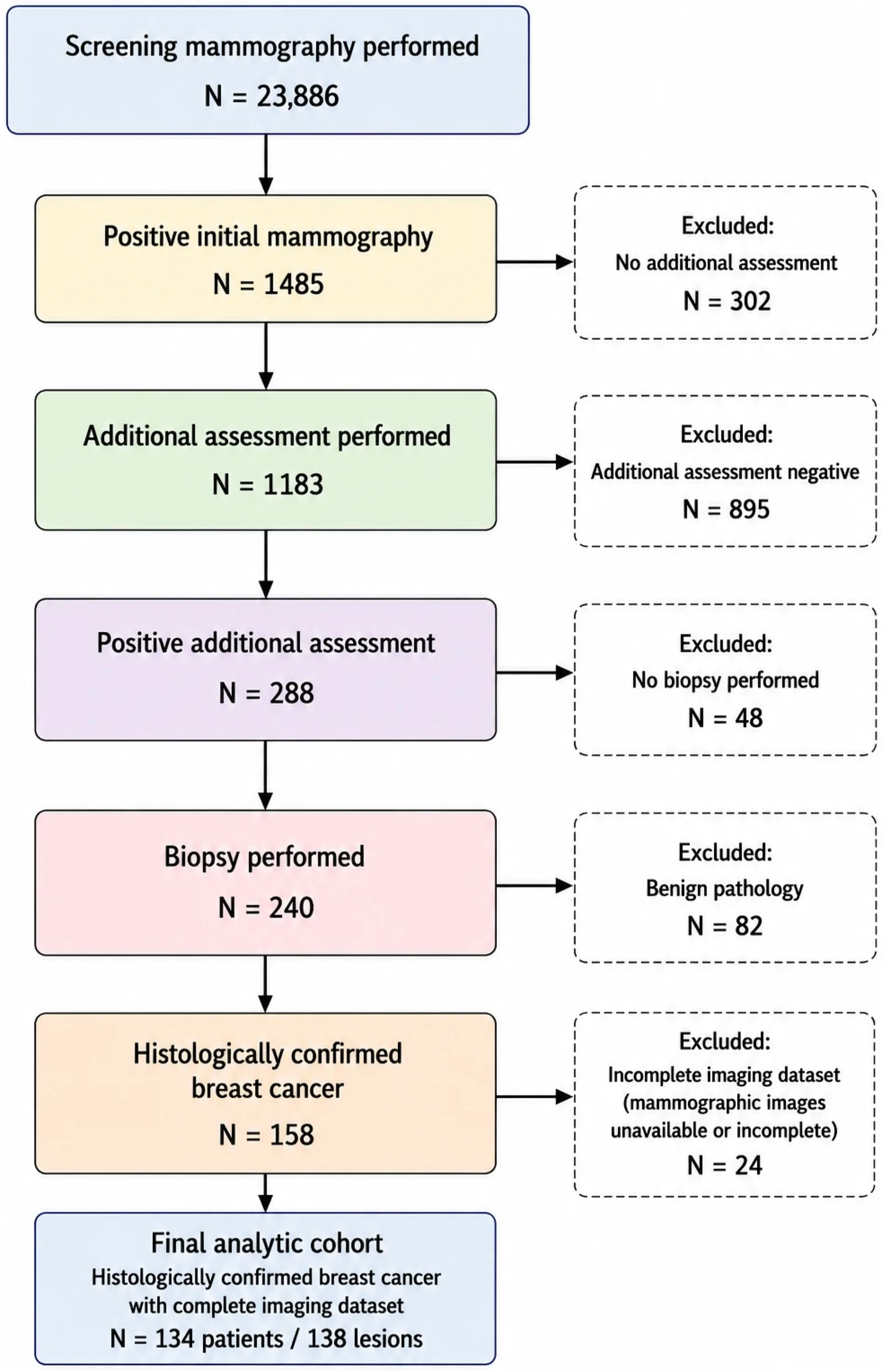
Flow-chart illustrating patient selection and study cohort formation within the regional breast cancer screening program. A total of 23,886 women underwent screening mammography. Cases requiring additional evaluation following screening mammography underwent targeted diagnostic workup and biopsy when indicated. After exclusion of benign histopathological results, the final analytic cohort included 134 patients with histologically confirmed breast cancer, corresponding to 138 breast lesions.

A total of 23,886 women underwent screening mammography during the study period. The participant selection process and diagnostic workflow, from initial screening mammography to histopathological confirmation, are summarized in the study flow chart (Figure 1). Women requiring additional assessment following screening mammography (BI-RADS 0, 4, or 5) [14] underwent further diagnostic evaluation, including targeted breast ultrasound and biopsy when indicated. Only histopathologically confirmed malignant lesions were included in the final analytic cohort.

The inclusion criteria were: (1) women aged between 50 and 69 years; (2) residence in the North-Eastern or South-Eastern administrative regions of Romania; (3) absence of clinical symptoms suggestive of breast cancer at the time of screening; and (4) no personal history of breast cancer.

Residential environment was categorized as urban or rural according to the official address recorded on the participants’ national identity documents. Vulnerability status was defined according to the predefined social inclusion criteria established within the methodological framework of the Romanian regional breast cancer screening programme [15]. Women were classified as vulnerable if they met at least one of the predefined criteria. These included low income, unemployment or economic inactivity, lack of health insurance, receipt of minimum-income or family-support benefits, self-employment in agriculture, rural residence, Roma ethnicity, disability, single-parent family status, homelessness, previous or current institutional care, substance dependence, detention or probation, and exposure to domestic violence or human trafficking [15]. Rural residence was explicitly included among the predefined vulnerability categories; however, vulnerability status in the present analysis was based on the classification recorded in the ONCOFEM project database. The two variables were therefore strongly, but not completely, overlapping, as some urban women were classified as vulnerable and a small number of rural women were recorded as non-vulnerable. The methodological framework required at least 50% of the target population of the regional screening programmes to belong to vulnerable groups [15]. Within the ONCOFEM project specifically, the planned target population comprised 30,000 women, of whom at least 16,000 were required to belong to vulnerable groups [16].

The final analytic cohort included 134 patients with histopathologically confirmed breast cancer, corresponding to 138 malignant breast lesions. Lesion-based analysis was performed for imaging characteristics, whereas sociodemographic variables were analyzed at patient level.

### 2.3. Imaging Protocol and Equipment

Screening mammography examinations were performed using three high-resolution full-field digital mammography systems. Two fixed mammography units were located at the Regional Institute of Oncology (IRO) Iași Screening Center: a GE Senographe Pristina 3D system (GE Healthcare, Chicago, IL, USA) and a Siemens Mammomat Revelation system (Siemens Healthineers, Erlangen, Germany).

In addition, a mobile mammography unit equipped with a GE Senographe Pristina™ Mobile system was deployed throughout underserved rural areas to improve geographic accessibility to screening services and reduce disparities in access to dedicated breast imaging infrastructure.

Standard bilateral craniocaudal (CC) and mediolateral oblique (MLO) projections were acquired for all participants according to institutional screening protocols.

### 2.4. Image Interpretation and Lesion Assessment

A double-reading protocol was implemented for all screening mammograms. Each examination was independently interpreted by two experienced breast radiologists blinded to the interpretation of the other reader. In cases of discordant findings, final assessment was established by arbitration performed by a third senior breast radiologist [17,18].

For all suspicious lesions, the maximum lesion diameter was defined as the largest measurable axis identified on standard mammographic projections and was recorded in millimeters. Lesion focality was classified according to mammographic appearance as unifocal, multifocal, or multicentric. Multifocal disease was defined as the presence of two or more malignant lesions within the same breast quadrant, whereas multicentric disease referred to lesions located in different quadrants of the same breast.

### 2.5. Diagnostic Workup and Histopathological Confirmation

Patients requiring additional assessment after screening mammography underwent targeted breast ultrasound examination. Ultrasound-guided core needle biopsy (CNB) was performed for suspicious lesions using Bard^®^ biopsy systems (C. R. Bard, Inc., Franklin Lakes, NJ, USA), including both reusable automatic biopsy guns and disposable automatic biopsy devices equipped with 14-gauge needles. A minimum of three tissue samples were obtained for each lesion to ensure adequate histopathological evaluation.

### 2.6. Statistical Analysis

Statistical analysis was performed using XLSTAT 2025 software (Lumivero, Denver, CO, USA). Continuous variables were expressed as median values with 25th and 75th percentiles (P25–P75) and as mean ± standard deviation (SD), whereas categorical variables were presented as frequencies and percentages.

Given the non-normal distribution of tumor dimensions and the presence of outlier lesions, the Mann–Whitney U test was used to compare maximum tumor dimensions between groups. Comparisons of categorical variables, including residential distribution, vulnerability status, lesion focality, recall rate, cancer detection rate, and the proportion of tumors exceeding 20 mm, were performed using the Chi-square test or Fisher’s exact test when appropriate.

A *p*-value < 0.05 was considered statistically significant.

### 2.7. Disclosure of the Use of Generative Artificial Intelligence

Generative artificial intelligence tools were used exclusively for language editing assistance, manuscript structuring, and optimization of scientific terminology. All generated content was critically reviewed, edited, and validated by the authors, who take full responsibility for the integrity and accuracy of the final manuscript.

## 3. Results

### 3.1. General Characteristics of the Screening Cohort

During the study period (August 2020–December 2023), a total of 23,886 women underwent screening mammography within the ONCOFEM regional breast cancer screening initiative. The residential distribution of the screened population was relatively balanced, comprising 12,652 (53.0%) women from rural areas and 11,234 (47.0%) from urban environments. The mean age of the overall cohort was 57.3 years (SD ± 5.4), with no statistically significant age difference observed between urban and rural participants (*p* = 0.082).

Overall, 13,210 women (55.3%) were classified as socially vulnerable according to ONCOFEM inclusion criteria. A strong association was observed between vulnerability and rural residence, as 99.8% of rural participants were categorized as vulnerable compared with only 5.2% of women from urban areas (*p* < 0.001).

Overall participation was higher in the North-Eastern region (15,218 women; 63.7%) than in the South-Eastern region (8668 women; 36.3%), with a significant association between geographic region and residential environment (*p* < 0.001). Baseline sociodemographic characteristics of the screening cohort are summarized in Table 1.

### 3.2. Screening Performance Metrics: Urban–Rural Differences

The overall recall rate, defined as cases requiring additional diagnostic assessment following screening mammography (BI-RADS 0, 4, or 5), was 6.22% for the entire cohort. Urban participants demonstrated a significantly higher recall rate compared with rural women (7.75% vs. 4.85%, respectively; *p* < 0.001).

The Cancer Detection Rate (CDR) was also significantly higher in the urban subgroup, reaching 7.21 cancers per 1000 screened women, compared with 4.19 cancers per 1000 screened women in the rural population (*p* = 0.002).

**Figure 2:**
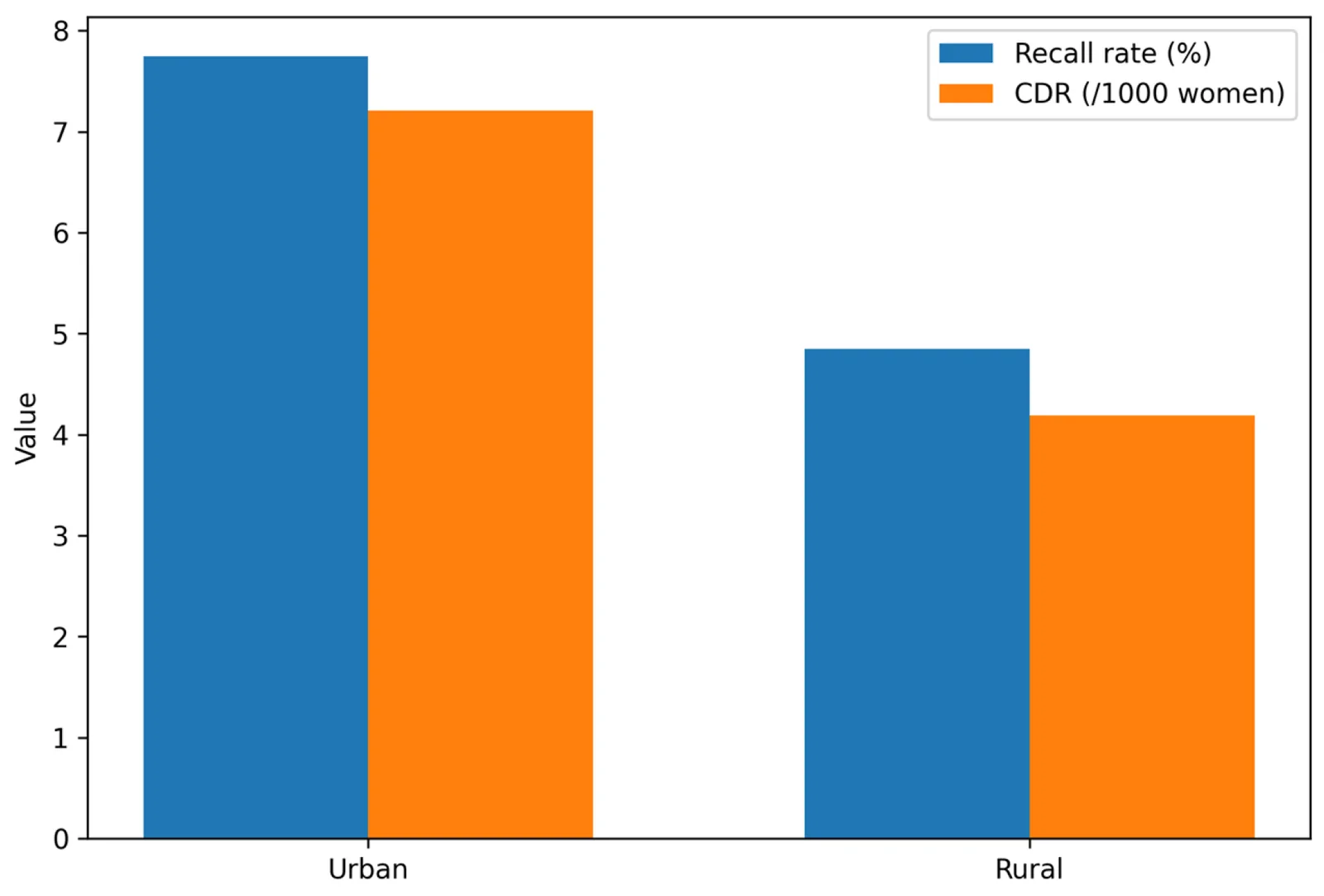
Comparison of screening performance metrics between urban and rural cohorts (*N* = 23,886). The grouped bar chart illustrates the recall rate (percentage of women referred for further assessment) and the cancer detection rate (number of confirmed malignant cases per 1000 women screened).

Figure 2 illustrates the comparison of recall rate and cancer detection rate between urban and rural screening populations.

### 3.3. Mammographic Tumor Burden at Diagnosis

The final analytic cohort included 138 histopathologically confirmed malignant breast lesions identified in 134 patients. The median maximum tumor dimension for the overall cohort was 25 mm (P25–P75: 17–37.5 mm), with a mean tumor size of 32.5 mm ± 24.3.

Although mean tumor size was higher in the urban subgroup, median tumor dimensions were similar between rural and urban populations, reflecting the presence of several high-dimensional outlier lesions. Rural patients demonstrated a numerically higher proportion of tumors exceeding 20 mm compared with urban participants (64.15% vs. 61.18%, respectively). However, no statistically significant difference in maximum tumor dimension was observed between urban and rural groups (*p* = 0.799).

Similarly, vulnerable women demonstrated a slightly higher median tumor dimension compared with non-vulnerable patients (27 mm vs. 25 mm, respectively), as well as a higher proportion of tumors exceeding 20 mm (64.91% vs. 60.49%). Nevertheless, these differences did not reach statistical significance (*p* = 0.934).

**Figure 3:**
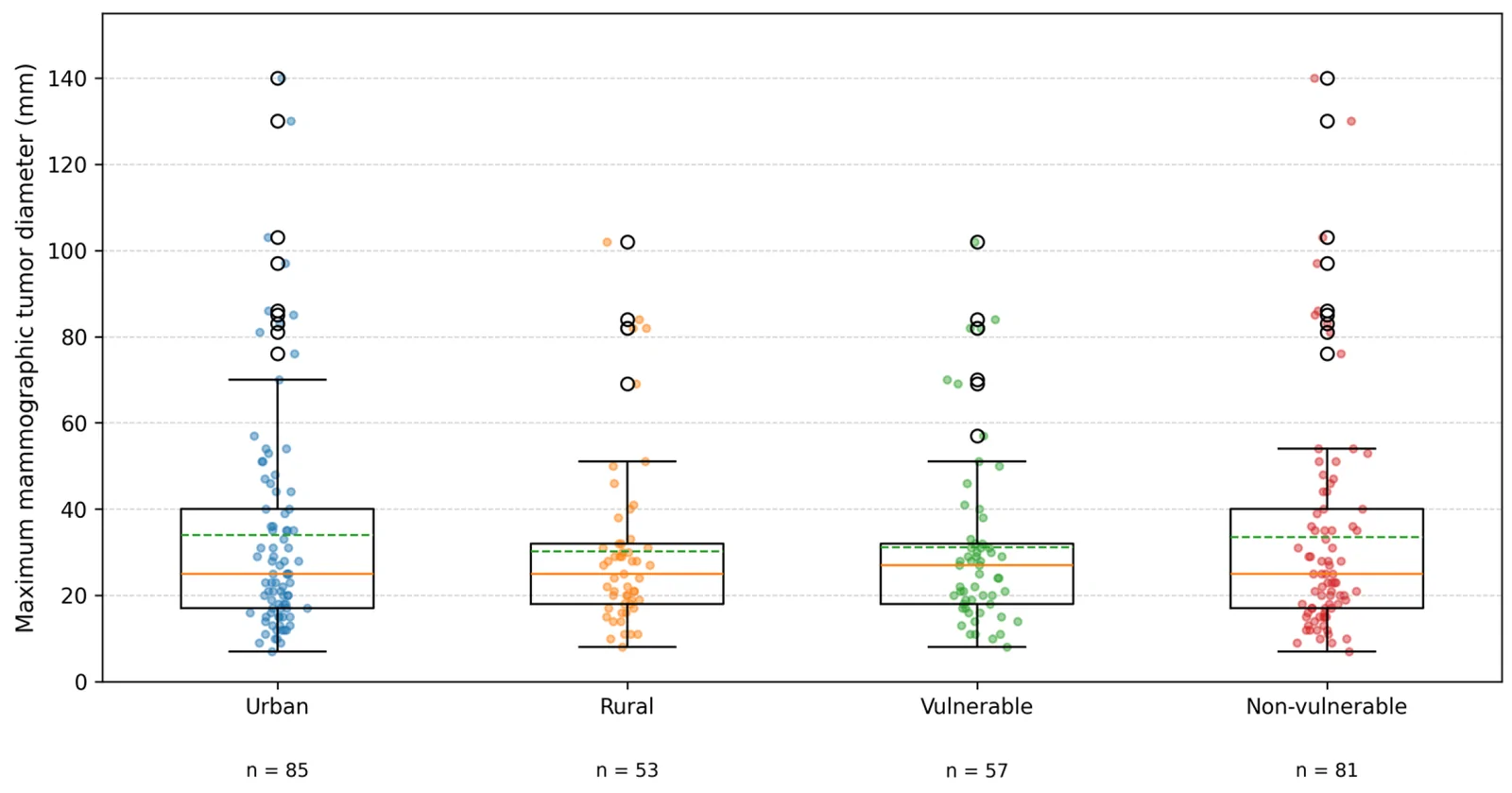
Distribution of maximum mammographic tumor diameter according to residential environment and socioeconomic vulnerability status. Boxplots represent median values, interquartile ranges, and outlier lesions.

Details regarding tumor dimensions and focality status are summarized in Table 2. Figure 3 illustrates the distribution of maximum tumor dimensions according to residential environment and vulnerability status.

Multifocal and multicentric disease represented a minority of cases across all analyzed subgroups. Unifocal lesions accounted for the majority of malignant tumors in both rural and urban populations, as well as in vulnerable and non-vulnerable groups. No statistically significant association was identified between sociodemographic subgroup and lesion focality pattern.

Similar proportions of large tumors were observed across all analyzed subgroups, ranging from 60.49% in non-vulnerable women to 64.91% in vulnerable patients. Overall, although significant urban–rural differences were observed in screening performance metrics, mammographic indicators of tumor burden demonstrated relatively similar distributions across the analyzed sociodemographic subgroups.

## 4. Discussion

This study evaluated the relationship between sociodemographic disparities and breast cancer burden at diagnosis within a large regional screening cohort from Eastern Romania, including 23,886 women screened between 2020 and 2023. While significant differences were identified in screening performance metrics between urban and rural populations, particularly regarding recall rate and cancer detection rate, no statistically significant associations were observed between residential environment or vulnerability and mammographic indicators of tumor burden at diagnosis.

One of the most important findings of the present study was the significantly higher recall rate and cancer detection rate observed among women from urban areas compared with rural participants. Several factors may contribute to this discrepancy. Urban populations generally benefit from improved access to healthcare infrastructure, greater health literacy, easier transportation to specialized medical centers, and increased familiarity with preventive healthcare programs [8,9,10]. In contrast, women from rural environments frequently encounter barriers related to geographic isolation, limited access to specialized breast imaging services, socioeconomic deprivation, and reduced participation in organized screening initiatives.

Despite these differences in screening performance, the imaging characteristics of confirmed malignant lesions demonstrated relatively comparable distributions across the analyzed sociodemographic subgroups. Although rural and vulnerable patients showed numerically higher proportions of tumors exceeding 20 mm, these differences did not reach statistical significance. Similarly, multifocal and multicentric disease represented only a minority of cases, without significant variation according to residential environment or vulnerability status.

Mobile screening systems were specifically introduced to improve accessibility for women living in geographically isolated rural areas, where access to dedicated breast imaging infrastructure remains limited [13]. Although no statistically significant differences in mammographic tumor burden were observed between the analyzed sociodemographic groups, the present study design does not allow this finding to be attributed to the implementation of mobile mammography. Further studies are required to determine whether improved geographic access through mobile screening translates into reduced urban–rural disparities in tumor burden at diagnosis.

Previous studies conducted in both Western and Eastern Europe have consistently demonstrated associations between socioeconomic disadvantage and more advanced breast cancer presentation at diagnosis [8,9,10]. Lower participation in screening programs has frequently been linked to larger tumors, delayed diagnosis, and poorer oncologic outcomes [8,9,10]. However, most available data originate from healthcare systems with long-established national screening infrastructures. Romania historically lacked a fully implemented nationwide breast cancer screening program, and therefore data regarding the interaction between social vulnerability and imaging-detected tumor burden remain limited.

An additional observation from the present study is the relatively high proportion of tumors exceeding 20 mm across all analyzed subgroups. This finding may reflect the characteristics of first-round population screening in a region without longstanding organized screening traditions, where a substantial proportion of detected cancers likely represent prevalent rather than incident lesions [19]. Consequently, larger tumor dimensions at diagnosis may still occur even within screened populations, particularly during the early implementation phases of organized screening initiatives.

The present study has several limitations. First, its retrospective design may introduce selection bias and limits the ability to establish causal relationships between sociodemographic variables and tumor characteristics. In addition, recruitment was invitation-based but capacity-limited and did not include all eligible women in the covered regions, which may have introduced selection bias and prevented the estimation of a population-level screening uptake rate. Second, despite the large overall screening cohort, the number of histopathologically confirmed malignant lesions included in the final analysis was relatively small (138 lesions in 134 patients), potentially limiting the statistical power to detect modest differences between sociodemographic subgroups, particularly regarding multifocal and multicentric disease. Third, vulnerability status and rural residence were strongly related because rural residence was explicitly included among the predefined vulnerability categories in the screening methodology. However, the variables were not completely overlapping in the ONCOFEM database, as some urban participants were classified as vulnerable and a small number of rural participants were recorded as non-vulnerable. This strong association limited the possibility of reliably separating their independent effects on mammographic tumour burden. Finally, tumor burden assessment relied primarily on mammographic lesion dimensions rather than complete pathological TNM staging.

Nevertheless, this study also presents several important strengths. To our knowledge, this represents one of the largest imaging-based analyses evaluating the relationship between sociodemographic disparities and breast cancer burden within an organized regional screening initiative in Romania. The study additionally reflects real-world implementation of a population-based screening strategy integrating double reading protocols, arbitration, dedicated breast imaging equipment, and mobile mammography services targeting underserved communities.

Overall, no statistically significant differences in mammographic tumor burden were observed between the analyzed sociodemographic groups; however, the present study design does not allow these findings to be interpreted as evidence that mobile mammography reduced pre-existing disparities.

Continued expansion of organized screening infrastructure, particularly in underserved regions, may play an important role in improving equity of access to early breast cancer detection in Eastern Europe.

## 5. Conclusions

This study evaluated the association between sociodemographic disparities and breast cancer burden at diagnosis within a large regional screening cohort from Eastern Romania. Although significant urban–rural differences were observed in screening performance metrics, including recall rate and cancer detection rate, no statistically significant associations were identified between residential environment or vulnerability and mammographic indicators of tumor burden at diagnosis. Nevertheless, a relatively high proportion of tumors exceeding 20 mm was observed across all analyzed subgroups, highlighting the persistent burden of relatively advanced disease at diagnosis within the Romanian population. These findings indicate that no statistically significant disparities in mammographic tumor burden were identified across the analyzed sociodemographic subgroups; however, the present study cannot determine whether the use of mobile mammography units contributed to this observation. Continued expansion of accessible population-based breast cancer screening programs may play an important role in improving the equity of early cancer detection in Romania and other Eastern European healthcare systems.

## Figures and Tables

**Table 1:** Baseline socio-demographic characteristics of the screening cohort (*N* = 23,886).

Characteristic	Total Population (*N* = 23,886)	Urban (*n* = 11,234)	Rural (*n* = 12,652)	*p*-Value
**Mean age, years ± SD**	57.3 ± 5.4	57.2 ± 5.5	57.4 ± 5.3	0.082
**Residence,** ***n*** **(%)**	23,886 (100%)	11,234 (47.0%)	12,652 (53.0%)	-
**Vulnerability Status,** ***n*** **(%)**				<0.001
**Vulnerable**	13,210 (55.3%)	585 (5.2%)	12,625 (99.8%)	
**Non-vulnerable**	10,676 (44.7%)	10,649 (94.8%)	27 (0.2%)	
**Region,** ***n*** **(%)**				<0.001
**North-East**	15,218 (63.7%)	6890 (61.3%)	8328 (65.8%)	
**South-East**	8668 (36.3%)	4344 (38.7%)	4324 (34.2%)	

**Table 2:** Mammographic tumor characteristics stratified by residence and vulnerability (*N* = 138 lesions).

Variable	Rural (*n* = 53)	Urban (*n* = 85)	*p*-Value	Vulnerable (*n* = 57)	Non-Vulnerable (*n* = 81)	*p*-Value
**Max Tumor Dimension (mm), mean ± SD**	30.25 ± 20.1	33.98 ± 26.6		31.12 ± 20.5	33.54 ± 26.7	
**Max Tumor Dimension (mm), median (P25–P75)**	25.0 (18–32)	25.0 (17–40)	0.799	27.0 (18–32)	25.0 (17–40)	0.934
**Tumor Size > 20 mm,** ***n*** **(%)**	34 (64.15%)	52 (61.18%)	0.726	37 (64.91%)	49 (60.49%)	0.598
**Focality Status,** ***n*** **(%)**			0.264			0.551
**Unifocal**	48 (90.6%)	72 (84.7%)		50 (87.7%)	70 (86.4%)	
**Multifocal**	5 (9.4%)	9 (10.6%)		7 (12.3%)	7 (8.6%)	
**Multicentric**	0 (0.0%)	4 (4.7%)		0 (0.0%)	4 (4.9%)	

## Data Availability

The data presented in this study are available from the corresponding author upon reasonable request. The data are not publicly available due to privacy and ethical restrictions related to the protection of participants’ personal and medical information.

## Abbreviations

The following abbreviations are used in this manuscript:

BI-RADS	Breast Imaging Reporting and Data System
CC	Craniocaudal
CDR	Cancer Detection Rate
CNB	Core Needle Biopsy
IRO	Regional Institute of Oncology
MLO	Mediolateral Oblique
SD	Standard Deviation
XLSTAT	Addinsoft XLSTAT Statistical Software
